# The Early Diagnosis and Treatment of Deformities of the Spine

**Published:** 1899-07-15

**Authors:** J. Jackson Clarke

**Affiliations:** Surgeon to Out-patients at the City Orthopædic and North-West London Hospitals


### Hospital Clinics and Medical Progress.

### THE EARLY DIAGNOSIS AND TREATMENT OF DEFORMITIES OF THE SPINE.

Abstract of a paper read before the North-West District of the Metropolitan Counties Branch of the British Medical Association by J. Jackson Clarke, M.B.Lond., F.R.C.S., Surgeon to Out-patients at the City Orthopædic and North-West London Hospitals.

(*Concluded from page* 243.)

**Scoliosis.**—Exhibiting a typical case of scoliosis, Mr. Jackson Clarke continued :—This patient affords an instance of right dorsal-convex scoliosis, with secondary left lumbar convex curve. Looking at the patient's back there appears at first sight to be no marked lateral deviation of the series of spinous processes, but the right scapula is more prominent than the left, and the right ribs are more prominent about their angles. There is a flattening to the left of the spine in the dorsal region which causes the lower angle of the left scapula to appear to stand out. The right shoulder is higher than the left. In the lumbar region the muscles on the left of the spine are more prominent than those on the right, owing to the rotation of the vertebræ to the right. On carefully marking out the spines, some deviation to the right in the upper and to the left in the lower part of the spine will be observed. The front of the chest shows undue prominence on the left and flattening on the right side. Viewed sideways, the normal antero-posterior curves may be either increased or diminished, or even reversed. In left dorsal-convex cases the condition is the counterpart, or, as it has been expressed, the "mirror-picture" of that described above. In certain cases the whole spine is involved in a lateral deviation to one or other side. In the beginning of the deformity only part of the spine may be involved; this is termed the "primary deviation." During the development of the deformity the patient usually complains of aching pain in the spine.

*Treatment.*—In deciding on the course of treatment to be adopted the stages of the affection are to be remembered. During the stage of development abnormal postures are to be corrected; the patient's diet, dress, &c., are to be regulated; fresh air and exercise short of fatigue must be provided for. The bed used by the patient should be firm—preferably a hair mattress. The tone of the muscles should be improved by massage and resisted exercises when necessary, and by carefully thought-out active exercises when the patient is sufficiently strong for them. Nothing is of more importance than to keep up the muscular tone in scoliosis. At this period, these cases often require instrumental support. Many instruments are worse than useless, and the best may do harm if they are wrongly adjusted. Instrumental treatment should be accompanied by regular exercises. By these means farther increase of deformity can be prevented, and in early cases and growing children the deformity can be cured. In cases of old standing Nature will often bring about an arrest of the deformity, and if the muscular tone is good no treatment may be required.

**Tuberculous Disease of the Spine.**—Destruction of one or more vertebral bodies necessitates a forward bend of the spine more or less pronounced. In the cervical and lumbar regions the first objective sign of the disease may be not an angular deformity, but a diminution of the normal concavity backwards. More or less pain is usually complained of, but this may be but little marked. In severe cases a "girdle-pain" is complained of. Tenderness is evidenced by the effect of shocks or jars upon the patient, as in taking a false step. Rigidity is a valuable symptom, and it may be elicited by requesting the patient to pick up some small object from the ground. Any part of the spine from the atlas to the coccyx may be involved, and the affection may show itself at any age from a few months to old age.

*Treatment.*—In an average early case free from complication the indications are—(1) To relieve pressure between the diseased bony surfaces; (2) to fix the spine in an improved position; and (3) to attend to the general health, spirits, and comfort of the patient. Many different methods are in use, and in different cases I have tried most of them. None has given me so good results as the use of a Chance's splint, combined with the prone couch. The splint consists of two steel uprights fixed to a broad pelvic band below, and to a pad from which soft straps pass over the clavicles and under the arms to be attached to the uprights below. In high dorsal and cervical cases a head-piece is added. Padded plates are fitted to the uprights at the seat of deformity, and rest upon the transverse processes (not upon the spines). These plates constitute fulcra, from which leverage is exerted by the shoulder-straps. By the use of the prone couch the patient is able to play with toys, or read according to his or her years. And it is of much importance to keep the patient occupied and interested during the day. If the case does well the patient may be allowed to walk after from eight to twelve months' of this treatment still wearing the support. In cases of older date the patient may be allowed to walk from the first. Fresh air, liberal diet, and cod-liver oil are also required.

**Rheumatoid Arthritis of the Spine** is often a more painful affection than tuberculosis. Neuralgic pain, tenderness, and deformity are the chief symptoms. The deformity may take the form of a general kyphosis. In one remarkable case of this kind that Sir William Broadbent recently sent to me the yielding of the vertebræ had been so rapid that paraplegia had developed. At other times scoliosis results. In the later stages great rigidity ensues. This affection may also begin at any age from early infancy to old age. In two patients now under my care the affection began before the age of four years. Proper diet, light support, and, later, gentle resisted exercises are called for in this affection, and in the vast majority of cases treatment is highly successful.

**Rickets** has already been mentioned in connection with scoliosis. The typical rachitic spinal deformity is a C-shaped backward curve. In infants Mr. Adams's wicker cradle, in children the moulded leather backboard, with massage, proper diet, and medicine are called for. It is to be remembered that in rickets the presence of adenoids, which in my opinion are usually part of the disease, tends to increase the deformity of the spine, and also to cause pigeon-breast and other thoracic deformities.

